# GYY4137 Regulates Extracellular Matrix Turnover in the Diabetic Kidney by Modulating Retinoid X Receptor Signaling

**DOI:** 10.3390/biom11101477

**Published:** 2021-10-07

**Authors:** Subir Kumar Juin, Sathnur Pushpakumar, Utpal Sen

**Affiliations:** Department of Physiology, University of Louisville School of Medicine, Louisville, KY 40202, USA; s0juin01@louisville.edu (S.K.J.); sbpush01@louisville.edu (S.P.)

**Keywords:** GYY4137, plasminogen activator inhibitor-1, retinoid X receptor, retinoic acid receptor, extracellular matrix, diabetic kidney, mesangial cells

## Abstract

Diabetic kidney is associated with an accumulation of extracellular matrix (ECM) leading to renal fibrosis. Dysregulation of retinoic acid metabolism involving retinoic acid receptors (RARs) and retinoid X receptors (RXRs) has been shown to play a crucial role in diabetic nephropathy (DN). Furthermore, RARs and peroxisome proliferator-activated receptor γ (PPARγ) are known to control the RXR-mediated transcriptional regulation of several target genes involved in DN. Recently, RAR and RXR have been shown to upregulate plasminogen activator inhibitor-1 (PAI-1), a major player involved in ECM accumulation and renal fibrosis during DN. Interestingly, hydrogen sulfide (H_2_S) has been shown to ameliorate adverse renal remodeling in DN. We investigated the role of RXR signaling in the ECM turnover in diabetic kidney, and whether H_2_S can mitigate ECM accumulation by modulating PPAR/RAR-mediated RXR signaling. We used wild-type (C57BL/6J), diabetic (C57BL/6-*Ins2^Akita^*/J) mice and mouse mesangial cells (MCs) as experimental models. GYY4137 was used as a H_2_S donor. Results showed that in diabetic kidney, the expression of PPARγ was decreased, whereas upregulations of RXRα, RXRβ, and RARγ1 expression were observed. The changes were associated with elevated PAI-1, MMP-9 and MMP-13. In addition, the expressions of collagen IV, fibronectin and laminin were increased, whereas elastin expression was decreased in the diabetic kidney. Excessive collagen deposition was observed predominantly in the peri-glomerular and glomerular regions of the diabetic kidney. Immunohistochemical localization revealed elevated expression of fibronectin and laminin in the glomeruli of the diabetic kidney. GYY4137 reversed the pathological changes. Similar results were observed in in vitro experiments. In conclusion, our data suggest that RXR signaling plays a significant role in ECM turnover, and GYY4137 modulates PPAR/RAR-mediated RXR signaling to ameliorate PAI-1-dependent adverse ECM turnover in DN.

## 1. Introduction

Diabetic nephropathy (DN) is the leading cause of chronic kidney disease (CKD) and end-stage renal disease (ESRD) [1,2,3,4,5]. The pathogenesis of DN is manifested by progressive glomerular and tubulointerstitial fibrosis, which is characterized by the excessive accumulation and deposition of extracellular matrix (ECM) proteins, leading to mesangial expansion and thickening of the glomerular basement membrane [2,6]. Renal fibrosis causes progressive decline of renal function and ultimately leads to ESRD. Matrix metalloproteinases (MMPs) are a family of zinc-dependent endopeptidases that regulate synthesis and degradation of the ECM [7,8]. An alteration in MMPs, such as MMP-9 and MMP-13, results in the disruption of synthesis and degradation of ECM proteins, leading to adverse ECM remodeling in DN [9,10,11,12,13]. Another important regulator of ECM is the serine protease plasminogen–plasmin system [14,15]. The primary physiological inhibitor of this system includes plasminogen activator inhibitor-1 (PAI-1) [15]. PAI-1 inhibits plasminogen activators that convert plasminogen to plasmin which, in turn degrades a wide range of ECM proteins including fibronectin and laminin [15,16]. Therefore, PAI-1 is instrumental in regulating ECM remodeling in the kidney [17,18]. PAI-1 is upregulated in DN [15,18,19,20,21,22,23,24]. PAI-1 deficiency and mutations have been shown to reduce renal fibrosis in experimental nephritis, unilateral ureteric obstruction (UUO) and DN [25,26,27]. Furthermore, PAI-1 is reported to be upregulated in hyperglycemia-induced mesangial cells [28,29].

The pleiotropic biological functions of vitamin A metabolite, i.e., retinoic acid, in the modulation of cell proliferation and differentiation, are regulated by the activation of two classes of nuclear receptors: retinoic acid receptors (RARα, β and γ) and retinoid X receptors (RXRα, β and γ) [30]. RARs respond to the all-trans retinoic acid (tRA) and 9-cis-isomers of retinoic acid, whereas RXRs are exclusively activated by the 9-cis-isomers of retinoic acid [31,32]. tRA is reported to inhibit the development of type-1 diabetes [33], and reduces body weight as well as adiposity by modulating lipid metabolism in mice [34,35,36]. RXR agonists act as insulin sensitizers that decrease hyperglycemia, hypertriglyceridemia and hyperinsulinemia, suggesting an anti-diabetic effect in type-2 diabetic mouse models [37]. Although RAR-mediated RXR signaling plays a critical role in the regulation of glucose and lipid metabolism, the role of RAR and RXR in the regulation of ECM turnover in DN remains underexplored. Pharmacological manipulation of retinoids as a potential anti-fibrotic agent in mesangial cells showed much promise in the treatment of CKD [38]. In contrast, in recent years, RAR- and RXR-mediated pro-fibrotic responses of retinoids have been observed in experimental CKD models [14,39]. However, the precise molecular mechanism behind their pro-fibrotic effects is still unclear. The peroxisome proliferator-activated receptor γ (PPARγ) is another ligand-activated transcription factor that belongs to the steroid/thyroid nuclear receptor superfamily. In the pathogenesis of diabetes, downregulation of PPARγ expression is associated with excessive matrix protein accumulation and glomerulonephritis [40,41,42]. The applications of synthetic ligands and agonists of PPARγ have emerged as promising treatment strategies to mitigate the progression of DN [43,44,45,46]. RARs and PPARs heterodimerize with RXR and the resulting heterodimer preferentially binds to specific PPAR-responsive elements (PPREs) on promoters to regulate transcription of the target genes [37,47,48]. RXR- and PPARγ-specific ligands selectively bind and activate RXR–PPARγ heterodimers but not RXR–RAR heterodimers [37,47,49]. PPARγ plays a crucial role in adipogenesis and acts as a major target for anti-diabetic agents [37,49]. Instead of monotherapy with RXR agonists, the combination treatment of RXR and PPARγ agonists was shown to be more effective in increasing insulin sensitization and reducing hyperglycemia in murine models of obesity and non-insulin-dependent diabetes mellitus [37].

Previous studies reported that DN is associated with a decrease in plasma H_2_S and deficiency in H_2_S, producing enzymes in the kidney [2]. These changes were found to be associated with ECM deposition and renal fibrosis in rat models of obstructive nephropathy, whereas a H_2_S donor inhibited renal fibrosis [50]. Recently, H_2_S was shown to protect against the development of DN by ameliorating renal fibrosis caused by excessive collagen deposition in an STZ-induced diabetic model [51]. Studies from our laboratory demonstrated that H_2_S deficiency results in adverse renovascular remodeling in hypertension and diabetes, whereas H_2_S supplementation maintains renal homeostasis by regulating MMPs and collagen [12,52,53,54]. A separate study showed that sodium hydrosulfide (NaHS) treatment prevents renal tubulointerstitial fibrosis by downregulating fibronectin expression in the obstructed murine kidney [55]. Previously, our group showed that H_2_S attenuates vascular calcification by upregulating elastin levels during hyperglycemia [56]. Recently, we demonstrated that GYY4137 (GYY), a H_2_S donor, mitigates adverse ECM remodeling by decreasing MMP-9 and MMP-13 expression in the kidney during type-1 DN and also regulates hypertensive ECM remodeling in mouse mesangial cells [13,57].

Although H_2_S has been shown to mitigate adverse renal complications of DN through ECM regulation, the precise signaling mechanism involved in diverse molecular events during ECM turnover remains unexplored. In the present study, we investigated the role of RXR signaling in the progression of DN and whether GYY mitigates ECM accumulation by the modulation of PPAR/RAR-mediated RXR signaling.

## 2. Materials and Methods

### 2.1. Animals and Experimental Groups

Male wild-type C57BL/6J mice (stock no. 000664) and diabetic C57BL/6-*Ins2^Akita^*/J mice (stock no. 003548) of 10–14 weeks old were obtained from the Jackson Laboratory (Bar Harbor, ME). Mice were fed standard mouse chow and water ad libitum, and were maintained under a 12:12 h light–dark cycle. All animal experiments were carried out in accordance with the institutional animal care and use committee-approved protocols (approval no. 20683, dated 2 December 2020) of the University of Louisville School of Medicine and conformed to the *Guide for the Care and Use of Laboratory Animals* of the National Institutes of Health (NIH Publication, 2011), U.S.A. Wild-type and diabetic Akita mice were treated either with normal saline or with GYY (0.25 mgKg^−1^ d^−1^, I.P.) for 8 weeks [13,58,59]. At the end of the experiment, mice were euthanized by using 2X tribromoethanol (TBE), and both blood and kidney samples were collected.

### 2.2. Antibodies and Reagents

Fibronectin (cat. no. ab2413), Laminin (cat. no. ab11575) and fluorescently conjugated anti-rabbit secondary antibodies (Alexa Fluor 488, cat. no. A-11008 and Alexa Fluor 594, cat. no. A-11012) were purchased from Abcam (Cambridge, CA, UK). PPARγ (cat. no. 2435S), RARγ1 (cat. no. 8965S), and PAI-1 (11907S) antibodies were from Cell Signaling Technology (Danvers, MA, USA). RXRα (cat. no. 21218-1-AP) antibody was from Proteintech (Rosemont, IL, USA). Collagen IV (cat. no. NBP1-26549) antibody was purchased from Novus Biologicals LLC (Centennial, CO, USA). MMP-9 (cat. no. MA5-15886) and MMP-13 (cat. no. 701287) antibodies were from Thermo Fisher Scientific (Waltham, MA, USA). RXRβ (cat. no. sc-293432), Elastin (cat. no. sc-58756), glyceraldehyde 3-phosphate dehydrogenase (GAPDH) (cat. no. sc-365062), and all HRP-conjugated secondary antibodies, i.e., anti-rabbit (cat. no. sc-2357), anti-mouse (cat. no. sc-516102), and anti-goat (cat. no. sc-2354) were purchased from Santa Cruz Biotechnology (Santa Cruz, CA, USA). GYY4137 (cat. no. SML0100), DAPI (cat. no. F6057), and other analytical reagents were from Sigma-Aldrich (St. Louis, MO, USA).

### 2.3. Isolation of RNA and Semi-Quantitative RT-PCR

Total RNA was extracted from cells and kidney using Trizol reagent (cat. no. 15596-026, Invitrogen, Carlsbad, CA, USA). The quality of total RNA was determined by NanoDrop ND-1000 and only highly pure RNA (260/280–2.00 and 260/230–2.00) was used for reverse transcriptase PCR (RT-PCR). Total RNA (1 μg) was reverse-transcribed using the EasyScript cDNA Synthesis kit (cat. no. G234, MidSci, St. Louis, MO, USA), following the manufacturer’s protocol. For the amplification of cDNA, PCR was performed using the GoTaq Hot Start Green Master Mix (cat. no. M5122, Promega, Madison, WI, USA), according to the manufacturer’s instructions. The PCR-amplified product was run on 1.5% agarose gel and visualized under UV light. The bands were quantified by densitometric analysis using ‘ImageJ’ software. The primer sequences (Invitrogen, Carlsbad, CA, USA) are listed in Table 1.

### 2.4. Cell Culture and Treatment

Mouse mesangial cells (MCs) were purchased from ATCC (Manassas, VA, USA). Cells were grown in a humidified 5% CO_2_ incubator at 37 °C in DMEM/F-12 50/50 medium containing 5% fetal bovine serum (ATCC, Manassas, VA, USA), antibiotics, and L-glutamine (Mediatech, Inc, Manassas, VA, USA). At 60% to 70% confluency, cells were seeded in 6-well culture plates at equal density (1 × 10^5^/well) containing glucose (5 mM, normal glucose, NG or 25 mM, high glucose, HG) followed by GYY (250 μM) treatment for 24 h [13,58,60]. 

### 2.5. Detection of H_2_S Production

H_2_S production by MCs was monitored by WSP-1, a reactive disulfide-containing fluorescent probe which selectively reacts with cellular H_2_S and generates fluorescence. Briefly, MCs were seeded onto the 12 mm cover slips and treated with glucose and GYY, as described above. After 24 h, culture media were removed, and the cells were incubated with 50 µM of WSP-1 (cat. no. 11179, Cayman Chemicals, Ann Arbor, MI, USA) in PBS; thereafter, the fluorescence of the cells was detected with a confocal microscope (Olympus IX80, Olympus Corporation, Tokyo, Japan).

### 2.6. Western Blotting

Protein was extracted from the kidney and mouse mesangial cells (MCs) by RIPA buffer (Boston BioProducts, Worcester, MA, USA) containing 1 mM phenylmethylsulfonyl fluoride (PMSF) and a 1% protease inhibitor cocktail (Sigma, St. Louis, MO, USA). After sonication, the protein lysate was centrifuged at 12,000× *g* at 4 °C for 10 min. Protein concentrations of the samples were determined by Bradford assay. An equal amount of protein extract (25 μg) was electrophoresed by SDS-PAGE and immunoblotted overnight onto the polyvinylidine difluoride (PVDF) membrane. The membranes were blocked with 5% non-fat dry milk in TBST for 1 h at room temperature, washed three times in TBST, and subsequently incubated overnight with the respective primary antibodies at 4 °C. Membranes were washed three times in TBST to remove the unbound primary antibody and then incubated with corresponding HRP-conjugated secondary antibodies for 2 h at room temperature. Thereafter, the membranes were washed and the immunoreactive protein bands were developed using ECL LuminataForte (Millipore, Temecula, CA, USA), visualized under a ChemiDoc MP System (Bio-Rad, Hercules, CA, USA) and quantified by densitometric analysis using ‘ImageJ’ software. 

### 2.7. Histological Collagen Staining

A ‘Masson trichrome stain kit’ (cat.no. 87019, Richard-Allan Scientific, Kalamazoo, MI, USA) was used to evaluate collagen deposition in kidney. Kidneys were fixed in neutral buffered formaldehyde solution for 24 h at room temperature, and thereafter, embedded in paraffin. 5 µm thick kidney sections were stained following the manufacturer’s protocol. The images were captured with an EVOS^®^ FL Automated System (Life Technologies, Inc., Grand Island, NY, USA) and analyzed using ‘ImageJ’ software.

### 2.8. Immunohistochemistry

The frozen kidneys were cryosectioned at 5 μm thickness and fixed with 4% paraformaldehyde for 20 min, followed by washing three times with PBST (0.1% Tween 20). Tissue sections were incubated with blocking buffer (1% BSA in PBST) for 1 h at room temperature, washed, and incubated with specific primary antibody overnight at 4 °C. After washing, the sections were incubated with the respective secondary antibodies conjugated with Alexa Fluor 488 and/or 594 (Invitrogen, Carlsbad, CA, USA) for 90 min at room temperature. After washing with PBST, images were captured by an Olympus FluoView1000 laser scanning confocal microscope (B&B Microscope, Pittsburgh, PA, USA) and analyzed using ‘ImageJ’ software. 

### 2.9. Statistical Analysis

Data are expressed as mean ± SD of three independent experiments or 6 mice/group. To determine the significance of differences between means of the different experimental groups, ANOVA was performed followed by Tukey’s post hoc test. *p* < 0.05 was considered to be significant.

## 3. Results

### 3.1. GYY Treatment Increased the Level of H_2_S in MCs under HG Condition

**Figure 1:**
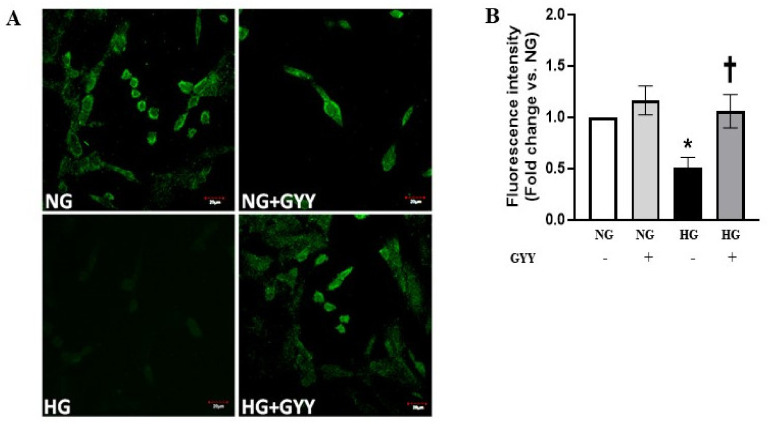
GYY treatment increased the level of H_2_S in MCs under HG condition. (**A**) Washington State Probe-1 (WSP-1), a reactive disulfide-containing fluorescent probe, was used to detect H_2_S in saline or GYY-treated mouse MCs in NG or HG conditions. (**B**) The bar graph represents the mean fold change ± SD in fluorescence intensity vs. NG. *n* = 3 independent experiments, * *p*  <  0.05 vs. NG, ^†^ *p*  <  0.05 vs. HG. Magnification, ×100; scale bar, 20 µm.

Excessive proliferation of mesangial cells (MCs) plays a crucial role in the development and progress of diabetic nephropathy (DN). Hydrogen sulfide (H_2_S) is an important gasotransmitter, which is reported to play a protective role in DN [6,61,62,63]. A recent study revealed that high glucose (HG) induces the overproliferation of mouse MCs by inhibiting H_2_S synthesis [62]. Our group previously showed that H_2_S donor GYY4137 (GYY) significantly increased the production of H_2_S in type-1 diabetic kidney. Therefore, we examined whether GYY treatment increases H_2_S production in MCs in HG condition. A significant decrease (49%) in H_2_S levels was observed in HG condition compared to that of an NG control (Figure 1A,B). GYY treatment restored normal H_2_S levels in HG conditions (Figure 1A,B). No significant change in H_2_S level was detected in NG condition with or without GYY treatment (Figure 1A,B).

### 3.2. Downregulation of PPARγ Expression Was Normalized by GYY Treatment in Diabetic Kidney and MCs under HG Condition

**Figure 2:**
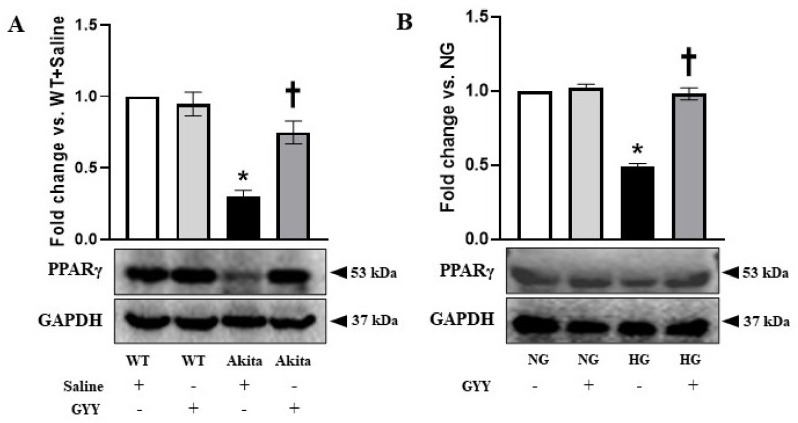
Downregulation of PPARγ expression was normalized by GYY treatment in diabetic kidney and MCs under HG condition. Protein was extracted from (**A**) saline- or GYY-treated kidneys from WT and Akita mice and (**B**) untreated or GYY-treated mouse MCs in NG or HG condition and analyzed for PPARγ expression by Western blot. The expression of PPARγ was normalized with GAPDH. The bar graphs represent the mean fold change ± SD vs. WT + Saline or NG. *n* = 6/group or 3 independent experiments, * *p*  <  0.05 vs. WT + Saline or NG, ^†^ *p*  <  0.05 vs. Akita + Saline or HG.

PPARγ, a member of the steroid/thyroid nuclear receptor superfamily, plays a critical role in glucose homeostasis [42,64,65]. In DN, PPARγ downregulation is associated with matrix accumulation [40,41,42]. Earlier studies demonstrated the efficacy of PPARγ agonists in the amelioration of DN [43,44,45,46,66]. Therefore, in the present study, we investigated the role of PPARγ in HG-exposed MCs as well as in the diabetic kidney and whether GYY has a regulatory role on PPARγ. Western blot analysis revealed that the expression of PPARγ was significantly decreased (70%) in the diabetic kidney compared to that of WT group (Figure 2A). GYY treatment significantly increased PPARγ level in the diabetic kidney (Figure 2A). However, PPARγ expression remained unaltered in the WT group following GYY treatment compared to that of the saline-treated WT control (Figure 2A).

Similarly, in the in vitro study, PPARγ expression was significantly decreased (51%) in MCs under HG condition compared to NG control, whereas GYY treatment normalized its expression comparable to NG group (Figure 2B). PPARγ expression was unaffected in the NG group by GYY (Figure 2B).

### 3.3. GYY Mitigated the Elevated Expression of RXRα, RXRβ and RARγ1 in Diabetic Kidney and MCs under HG Conditions

**Figure 3:**
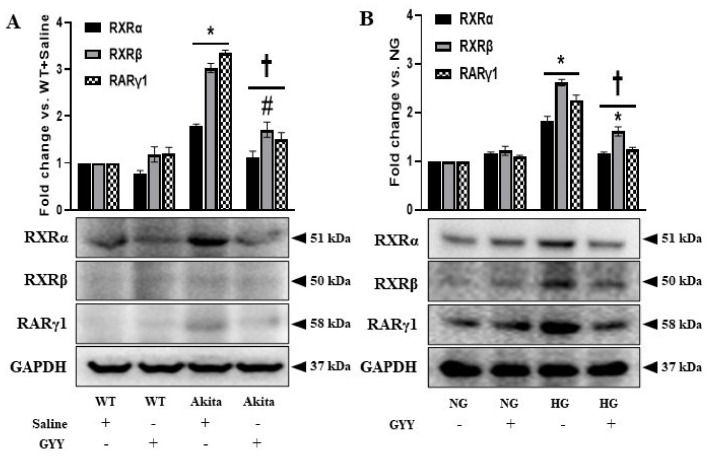
GYY-mitigated elevated expression of RXRα, RXRβ and RARγ1 in diabetic kidney and MCs under HG condition. Protein was extracted from (**A**) saline- or GYY-treated kidneys from WT and Akita mice and (**B**) untreated or GYY-treated mouse MCs in NG or HG condition and analyzed for the expression of RXRα, RXRβ and RARγ1 by Western blot. The expression of each protein was normalized with GAPDH. The bar graphs represent the mean fold change ± SD vs. WT + Saline or NG. *n* = 6/group or 3 independent experiments, * *p*  <  0.05 vs. WT + Saline or NG, ^†^ *p*  <  0.05 vs. Akita + Saline or HG, ^#^ *p* <  0.05 vs. WT + GYY.

Earlier studies demonstrated the role of RAR/RXR-mediated signaling in the regulation of diabetes and the role of retinoids as potential anti-fibrotic candidates in mesangial cells [37,38]. Conversely, RXR- and RAR-mediated pro-fibrotic effects of retinoids have been elucidated in CKD models [14,39]. However, the role of RAR and RXR in ECM turnover in the diabetic kidney remains largely unknown. In the present study, we examined the expression of RXRα, RXRβ and RARγ1 in kidneys and mouse MCs and tested whether GYY modulates their expression. The expressions of RXRα, RXRβ and RARγ1 were not changed significantly in WT and NG controls following GYY treatment compared to the basal level expression of respective controls (Figure 3A,B). The expressions of RXRα, RXRβ and RARγ1 were upregulated in diabetic kidney (78%, 203% and 235%, respectively) and MCs under HG condition (83%, 162% and 126%, respectively) (Figure 3A,B). GYY treatment significantly reduced the levels of RXRα, RXRβ and RARγ1 in diabetic mice and MCs under HG condition compared to saline-treated diabetic mice and untreated MCs under HG condition, respectively (Figure 3A,B).

### 3.4. Upregulated PAI-1 Expression Was Normalized in Diabetic Kidney and MCs under HG Condition by GYY Treatment

**Figure 4:**
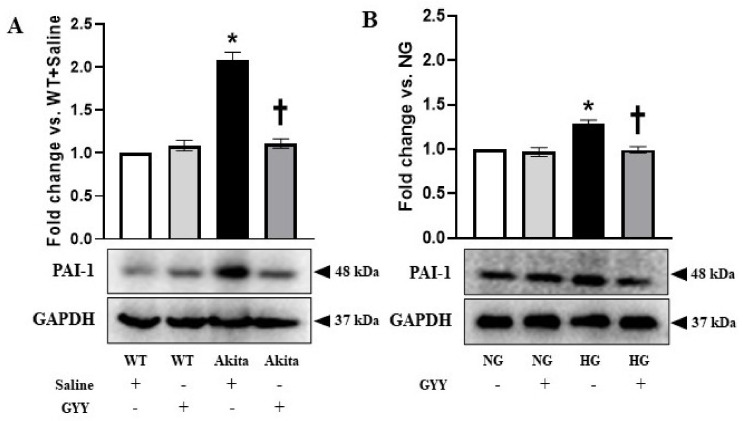
Upregulated PAI-1 expression was normalized in diabetic kidney and MCs under HG condition by GYY treatment. Protein was extracted from (**A**) saline- or GYY-treated kidneys from WT and Akita mice and (**B**) untreated or GYY-treated mouse MCs in NG or HG condition and analyzed for PAI-1 expression by Western blot. The expression of PAI-1 was normalized with GAPDH. The bar graphs represent the mean fold change ± SD vs. WT + Saline or NG. *n* = 6/group or 3 independent experiments, * *p*  <  0.05 vs. WT + Saline or NG, ^†^ *p*  <  0.05 vs. Akita + Saline or HG.

PAI-1 is an important regulator of ECM homeostasis [57,67]. Deregulation of PAI-1 has been evidenced in kidney fibrosis during hypertension and diabetes [23,57,67,68,69,70]. Therefore, in the current study, we investigated whether GYY treatment modulates the expression of PAI-1 in diabetic kidney and MCs under HG condition. The expression of PAI-1 in the nondiabetic control kidney as well as in MCs under NG condition was at basal level (Figure 4A,B). There was no significant change in PAI-1 expression in WT and NG control following GYY treatment (Figure 4A,B). In the diabetic kidney and MCs under HG condition, PAI-1 was upregulated by 109% and 29%, respectively, compared to the controls (Figure 4A,B). GYY treatment normalized PAI-1 expression in diabetic kidney and MCs under HG condition (Figure 4A,B). 

### 3.5. Elevated Expressions of MMP-9 and MMP-13 Were Alleviated by GYY Treatment in Diabetic Kidney and MCs under HG Condition

**Figure 5:**
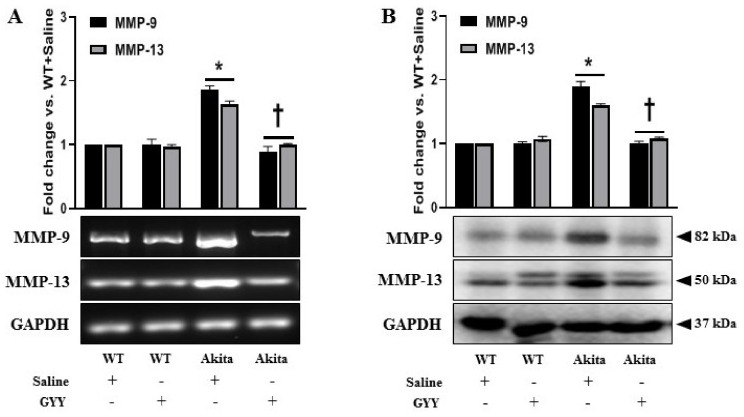
Elevated expressions of MMP-9 and MMP-13 were alleviated by GYY treatment in diabetic kidney. (**A**) Saline- or GYY-treated kidneys from WT and Akita mice were collected in Trizol for total RNA extraction, and semi-quantitative RT-PCR analyses were performed for MMP-9 and MMP-13 gene expression. (**B**) Protein was extracted from the saline- or GYY-treated kidneys from WT and Akita mice and analyzed for the expression of MMP-9 and MMP-13 by Western blot. GAPDH was used as a loading control for all RT-PCR and immunoblot analyses. The bar graphs represent the mean fold change ± SD vs. WT + Saline. *n* = 6/group, * *p*  <  0.05 vs. WT + Saline, ^†^ *p*  <  0.05 vs. Akita + Saline.

**Figure 6:**
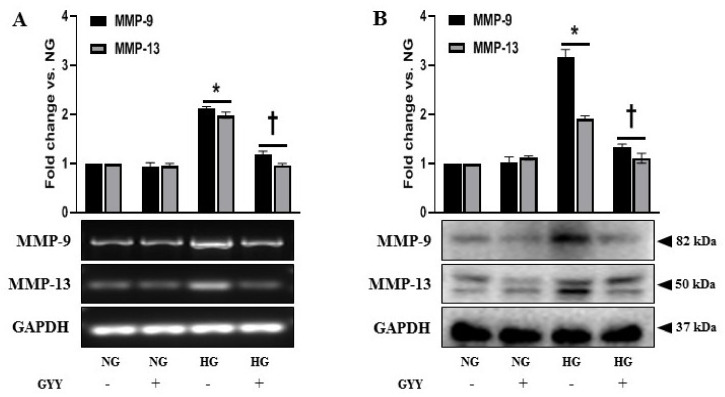
Elevated expressions of MMP-9 and MMP-13 were alleviated by GYY treatment in MCs under HG condition. (**A**) Untreated or GYY-treated mouse MCs in NG or HG condition were collected in Trizol for total RNA extraction, and semi-quantitative RT-PCR analyses were performed for MMP-9 and MMP-13 gene expression. (**B**) Protein was extracted from the untreated or GYY-treated mouse MCs in NG or HG condition and analyzed for the expression of MMP-9 and MMP-13 by Western blot. GAPDH was used as a loading control for all RT-PCR and immunoblot analyses. The bar graphs represent the mean fold change ± SD vs. NG. *n* = 3 independent experiments, * *p*  <  0.05 vs. NG, ^†^ *p*  <  0.05 vs. HG.

In DN, MMPs play crucial roles in the progression of renal fibrosis by disrupting the normal synthesis and degradation of ECM proteins [8,10,12,57,71]. Both MMP-9 and MMP-13 upregulation and a decrease in H_2_S are associated with diabetic renal remodeling [12,13,54,72]. Therefore, we examined the changes in MMP-9 and MMP-13 expression in response to GYY treatment in kidneys and MCs. The mRNA and protein expression of MMP-9 and MMP-13 in WT and NG controls were at basal levels that remained statistically unaltered following GYY treatment (Figure 5A,B). In diabetic kidney, MMP-9 and MMP-13 were increased at mRNA (86% and 64%, respectively) and protein (90% and 61%, respectively) levels compared to WT control (Figure 5A,B). Notably, the expression of MMP-9 was higher than MMP-13 at both mRNA and protein levels (Figure 5A,B). In the diabetic kidney, GYY treatment normalized MMP-9 and MMP-13 levels that were comparable to WT control (Figure 5A,B). Consistent with in vivo data, our in vitro results revealed no significant difference in mRNA and protein levels of MMP-9 and MMP-13 in MCs treated with or without GYY under NG conditions (Figure 6A,B). In HG-exposed MCs, MMP-9 and MMP-13 were upregulated in the mRNA (112% and 98%, respectively) and protein (217% and 92%, respectively) levels (Figure 6A,B). In agreement with the in vivo data, mRNA and protein expression of MMP-9 was higher compared to MMP-13 in MCs under HG. GYY treatment normalized the expression of MMP-9 and MMP-13 in MCs under HG conditions (Figure 6A,B).

### 3.6. GYY Ameliorated the Altered Expression of Collagen IV, Fibronectin, Laminin and Elastin in Diabetic Kidney and MCs under HG Condition

**Figure 7:**
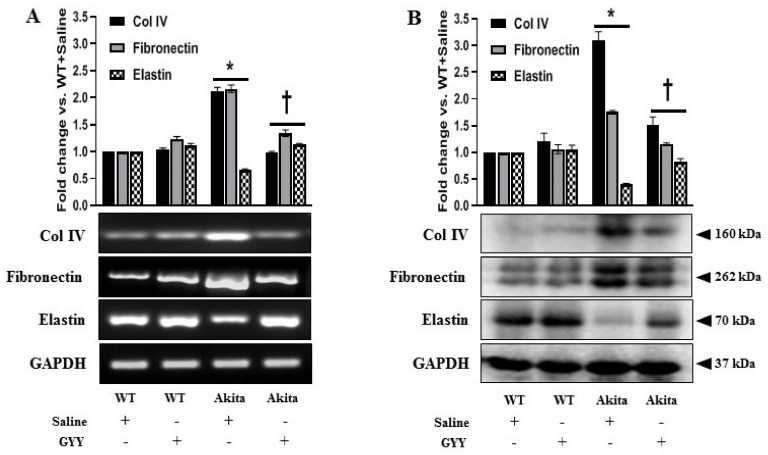
GYY ameliorated the altered expression of Col IV, fibronectin and elastin in diabetic kidney. (**A**) Saline- or GYY-treated kidneys from WT and Akita mice were collected in Trizol for total RNA extraction, and semi-quantitative RT-PCR analyses were performed for Col IV, fibronectin and elastin gene expression. (**B**) Protein was extracted from the saline- or GYY-treated kidneys from WT and Akita mice and analyzed for the expression of Col IV, fibronectin and elastin by Western blot. GAPDH was used as a loading control for all RT-PCR and Western blot analyses. The bar graphs represent the mean fold change ± SD vs. WT + Saline. *n* = 6/group, * *p*  <  0.05 vs. WT + Saline, ^†^ *p*  <  0.05 vs. Akita + Saline.

**Figure 8:**
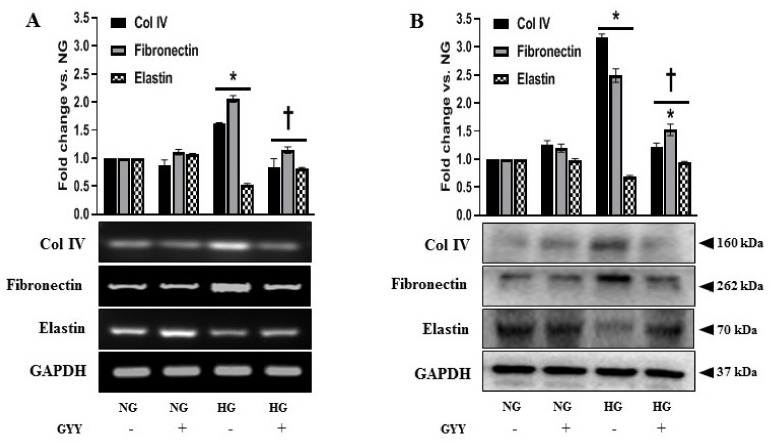
GYY ameliorated the altered expression of Col IV, fibronectin and elastin in MCs under HG condition. (**A**) Untreated or GYY-treated mouse MCs in NG or HG condition were collected in Trizol for total RNA extraction, and semi-quantitative RT-PCR analyses were performed for Col IV, fibronectin and elastin gene expression. (**B**) Protein was extracted from the untreated or GYY-treated mouse MCs in NG or HG condition and analyzed for the expression of Col IV, fibronectin and elastin by Western blot. GAPDH was used as a loading control for all RT-PCR and immunoblot analyses. The bar graphs represent the mean fold change ± SD vs. NG. *n*= 3 independent experiments, * *p*  <  0.05 vs. NG, ^†^ *p*  <  0.05 vs. HG.

Renal fibrosis is characterized by the excessive accumulation and deposition of ECM in the mesangium and tubulointerstitium, leading to a progressive decline in renal function during DN [1,2]. MCs are involved in the regulation of ECM accumulation in the pathogenesis of DN [2,73]. Therefore, we investigated the expression of collagen IV (Col IV), fibronectin, and elastin in the diabetic kidney and in MCs under HG stimulation and tested whether GYY modulated their expression. A significant increase in the expression of Col IV and fibronectin was observed in the mRNA (111% and 116%, respectively) and protein (210% and 76%, respectively) levels in the diabetic kidney compared to WT controls (Figure 7A,B). On the other hand, mRNA and protein expressions of elastin in diabetic kidney were significantly downregulated by 35% and 60%, respectively, compared to the control (Figure 7A,B). Similarly, a sharp upregulation of Col IV and fibronectin was evidenced both at mRNA (61% and 105%, respectively) and protein (217% and 149%, respectively) levels in the HG-exposed MCs compared to the NG control (Figure 8A,B). Elastin expression was significantly decreased, both at mRNA (47%) and protein (31%) levels in MCs under HG conditions compared to the NG control (Figure 8A,B). GYY treatment on Akita mice and HG-exposed MCs significantly reduced Col IV and fibronectin expression and increased elastin expression both at mRNA and protein levels compared to that of saline-treated diabetic mice and MCs under HG conditions, respectively (Figure 7A,B and Figure 8A,B). There were no significant differences in the mRNA and protein expression of Col IV, fibronectin and elastin between saline-treated and GYY-treated WT kidney and also between untreated and GYY-treated MCs under NG conditions (Figure 7A,B and Figure 8A,B).

**Figure 9:**
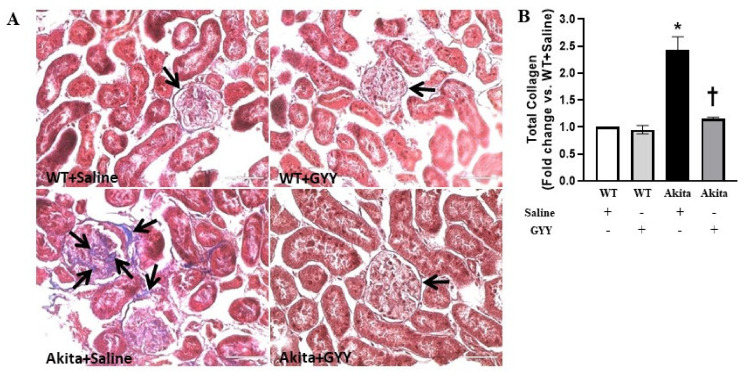
GYY reduced glomerular collagen deposition in diabetic kidney. (**A**) Representative photomicrograph of kidney section stained with Masson’s trichrome showing predominant collagen deposition at the peri-glomerular and glomerular regions (black arrows). (**B**) The bar graph represents the mean fold change ± SD of total collagen area vs. WT + Saline. *n* = 6/group, * *p*  <  0.05 vs. WT + Saline, ^†^ *p*  <  0.05 vs. Akita + Saline. Magnification × 60; scale bar: 50 µm.

**Figure 10:**
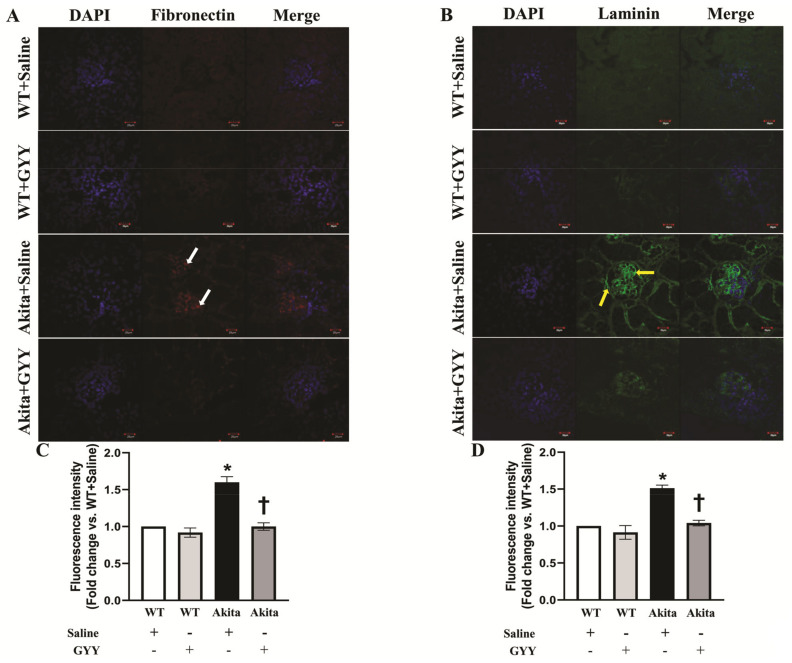
GYY mitigated the increased expression of fibronectin and laminin in diabetic kidney. Representative photomicrograph of kidneys immunostained with specific primary and fluorescent-tagged secondary antibodies against (**A**) fibronectin (red, white arrow) and (**B**) laminin (green, yellow arrow). The nuclei were counterstained with DAPI (blue). The bar graphs represent the mean fold change ± SD in fluorescence intensity vs. WT + Saline for (**C**) fibronectin and (**D**) laminin. *n* = 6/group, * *p*  <  0.05 vs. WT + Saline, ^†^ *p*  <  0.05 vs. Akita + Saline. Magnification, × 60; scale bar, 20 µm.

To determine the extent and regions of collagen deposition in the kidney, we stained kidney sections with Masson’s trichrome stain. Collagen accumulation was significantly increased in diabetic kidney, predominantly in the peri-glomerular and glomerular region compared to WT kidney (Figure 9A,B). GYY treatment reduced collagen accumulation to basal expression levels observed in WT kidney (Figure 9A,B). No significant difference was observed in collagen deposition in the glomeruli of saline-treated and GYY-treated WT kidneys (Figure 9A,B). Similar to the mRNA and immunoblot analyses, immunohistochemical staining revealed that fibronectin expression was substantially increased in the glomeruli of the diabetic kidney (Figure 10A,C). Interestingly, GYY treatment on Akita mice reduced the fibronectin expression to the basal level observed in WT kidney (Figure 10A,C). Fibronectin expression in the glomeruli of GYY-treated WT mice remained unchanged compared to that of saline-treated WT mice (Figure 10A,C). Furthermore, in the diabetic kidney, immunohistochemical localization showed a robust upregulation of laminin in the glomeruli of the diabetic kidney compared to that of WT mice (Figure 10B,D). The expression of laminin in the kidney section of diabetic mice that received GYY treatment remained at the basal level, which was comparable to WT mice (Figure 10B,D). However, laminin expression in the kidney section of WT mice treated with GYY was unchanged compared to saline-treated WT mice (Figure 10B,D).

### 3.7. ‘STITCH 5.0’ Protein–Protein Interaction Network Reinforced the Putative Involvement of RXR Signaling in the Regulation of PAI-1-Mediated ECM Turnover

**Figure 11:**
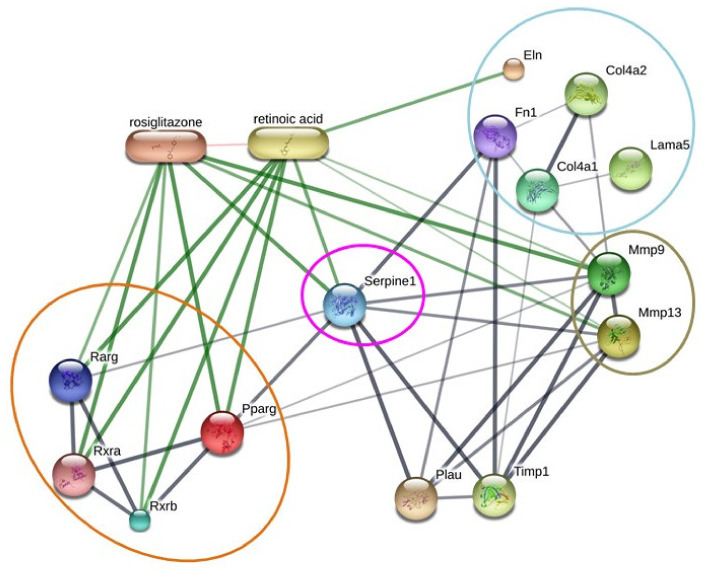
‘STITCH 5.0’ protein–protein interaction network reinforces the putative involvement of RXR signaling in the regulation of PAI-1-mediated ECM turnover. The stronger associations are represented by thicker lines. Protein–protein interactions are shown in grey, and chemical–protein interactions are depicted in green. The interactions between chemicals are exhibited in red. Pparg: peroxisome proliferator-activated receptor gamma; Rxra, retinoid X receptor alpha; Rxrb, Retinoid X receptor beta; Rarg, retinoic acid receptor gamma; Mmp9, matrix metallopeptidase 9; Mmp13, matrix metallopeptidase 13; Col4a1, collagen, type IV, alpha 1; Col4a2, collagen, type IV, alpha 2; Fn1, fibronectin 1; Lama5, laminin, alpha 5; Eln, elastin; Plau, plasminogen activator, urokinase; Timp1, tissue inhibitor of metalloproteinase 1.

‘STITCH 5.0’ protein interaction network analysis was employed to validate the principal findings of our study and to obtain an overview of the possible mechanistic insight of ECM turnover in the diabetic kidney. The analysis evidenced potential strong interactions of PPARγ and RARγ with RXRs (RXRα and RXRβ). However, PPARγ and RARγ showed a direct interaction with serpine 1 (alternatively known as PAI-1), which was found to be associated with ECM proteins, i.e., fibronectin, Col IV, and laminin, either directly or via MMP-9 and MMP-13 (Figure 11). Furthermore, PAI-1 exhibited direct and strong associations with fibronectin and MMP-9 and MMP-13. Taken together, the analysis reinforced the plausible involvement of PPAR/RAR-mediated RXR signaling in the modulation of PAI-1-dependent ECM turnover in diabetic kidney.

## 4. Discussion

**Figure 12:**
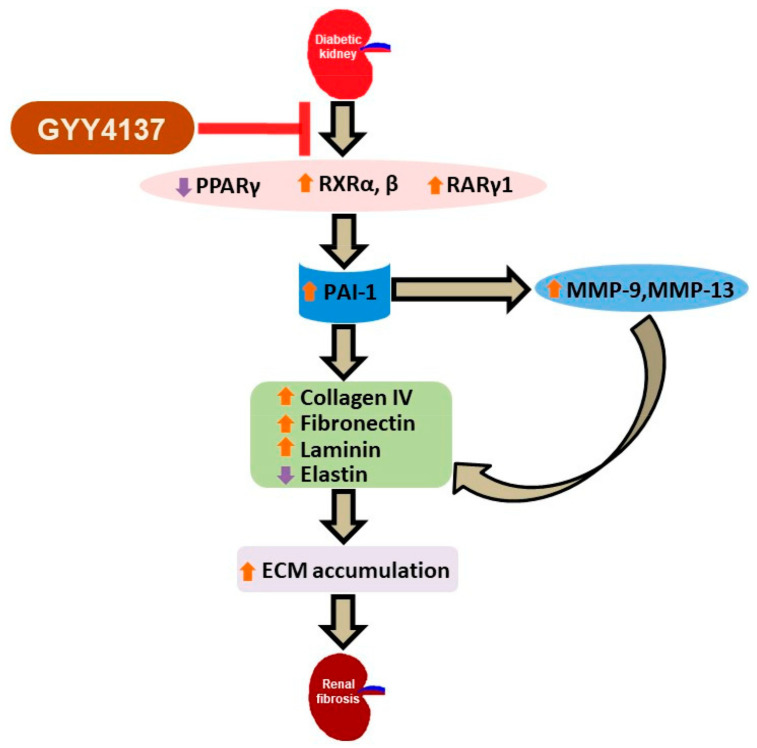
Schematic representation of the comprehensive findings. In diabetic kidney, hyperglycemia results in the downregulation of PPARγ and the upregulation of RXRα, RXRβ and RARγ1, which contribute to elevated PAI-1, MMP-9 and MMP-13 levels. The changes lead to the elevated expression of Col IV, fibronectin and laminin and decreased elastin expression, causing excessive ECM accumulation, leading to renal fibrosis in the diabetic kidney. Supplementation of an H_2_S donor, GYY, ameliorates adverse ECM remodeling by reversing the altered expression of PAI-1, MMP-9 and MMP-13 via the modulation of RXR signaling.

In diabetic nephropathy (DN), several metabolic anomalies contribute to pathogenesis, resulting in the progressive development of glomerular and tubulointerstitial fibrosis. Renal fibrosis is characterized by the excessive synthesis and deposition of ECM proteins leading to end-stage renal disease (ESRD) and kidney failure. In recent years, H_2_S has emerged as an important endogenous gasotransmitter that regulates various metabolic disorders including diabetic complications [6,74,75,76]. The reduction in endogenous H_2_S production in type-1 diabetic mice is a well-accepted paradigm. Several studies have revealed that a reduction in endogenous synthesis of H_2_S and H_2_S-producing enzymes are correlated with excessive ECM accumulation in the diabetic kidney, whereas the induction of H_2_S by exogenous administration ameliorates ECM remodeling [13,77,78]. However, mechanisms of H_2_S action in DN are not completely understood. The present study investigated the role of RXR signaling in excessive ECM accumulation in type-1 diabetic kidney and whether the exogenous supplementation of H_2_S can ameliorate adverse ECM turnover by modulating PPAR/RAR-mediated RXR signaling. Our findings revealed that in diabetic kidney, the downregulation of PPARγ and upregulation of RXRα, RXRβ and RARγ1 were associated with elevated levels of PAI-1 as well as MMP-9 and MMP-13. Increased PAI-1 and imbalance in MMPs resulted in the upregulation of Col IV, fibronectin and laminin, and the downregulation of elastin expression, leading to excessive ECM accumulation in the diabetic kidney. Supplementation of GYY ameliorated excessive ECM accumulation by reducing the expression of PAI-1 and MMPs via the modulation of PPAR/RAR-mediated RXR signaling. Hyperglycemia plays a central role in the development and progression of DN, which is characterized by an expansion of the glomerular mesangium, due to the overproliferation of glomerular mesangial cells and an excessive accumulation of ECM proteins synthesized by mesangial cells [5,79]. Therefore, we examined the potential regulatory role of GYY supplementation on RXR signaling in murine MCs exposed to HG. Our in vitro observations showed that HG-induced diminished H_2_S production by MCs gives rise to pathological responses similar to that of diabetic kidney, whereas GYY treatment substantially mitigated those adverse effects. The major findings and the possible regulatory pathway of GYY in ECM turnover in type-1 diabetic kidney are highlighted in Figure 12. 

Previous studies from our laboratory and others revealed a marked decrease in renal H_2_S production and plasma levels of H_2_S in Akita mice, whereas the supplementation of H_2_S increased both plasma and tissue levels of H_2_S [2,13,54,58]. In the present study, a significant decrease in endogenous production of H_2_S was detected in MCs exposed to HG conditions, whereas GYY treatment substantially increased H_2_S production by the MCs under HG conditions in vitro. These observations support previous in vitro studies [58,62,77]. Moreover, our in vitro results further support the previous in vivo findings that hyperglycemia decreases H_2_S levels, whereas the exogenous supplementation of H_2_S is able to restore normal levels in Akita mice [13,54,77]. Therefore, a therapeutic potential of GYY can be suggested to restore normal H_2_S levels in diabetic conditions. Although a negative correlation between hyperglycemia and H_2_S level has been demonstrated in both type-1 and type-2 diabetic conditions, the precise molecular mechanisms involved in the crosstalk between glucose metabolism and H_2_S production are yet to be determined [13,80,81].

The nuclear receptor PPARγ is an important regulator of glucose and lipid homeostasis [42,45,64,65]. Renoprotective effects of several PPARγ agonists (e.g., thiazolidinedione, ciglitazone, pioglitazone, troglitazone, rosiglitazone, etc.) are currently in use to reduce fibrosis and diabetic complications in the kidney [43,44,45,46,66]. Earlier studies revealed that H_2_S prevents hyperhomocysteinemia-induced renal failure by regulating MMPs and collagen in vivo [52,53]. Recently, the PPARγ agonist ‘ciglitazone’ was shown to ameliorate DN by decreasing a H_2_S precursor, i.e., homocysteine [82]. Therefore, PPARγ regulation by H_2_S may be an effective therapeutic approach in the treatment of DN. Our results showed that the downregulation of PPARγ in the diabetic kidney and HG-exposed MCs, corroborating previous findings [40,41,42]; GYY treatment restored normal levels by reversing the effect of hyperglycemia and HG. Previous reports showed that in DN, downregulated PPARγ expression is associated with matrix accumulation [40,41,42]. Together, the findings of our study suggest that PPARγ regulation by GYY may play a crucial role in modulating ECM accumulation in diabetic kidney.

ECM accumulation is mainly regulated by two pathways, i.e., the serine protease plasminogen–plasmin pathway and the MMP pathway [14]. PAI-1 inhibits the conversion of plasminogen activators to plasmin, which degrades matrix proteins either directly or through the activation of latent MMPs [14,15]. Therefore, PAI-1 and MMPs are central regulators of ECM remodeling in DN [8,10,12,18,57,67,71]. Our study revealed a sharp upregulation of PAI-1 in the diabetic kidney compared to WT controls, confirming the previously reported role of PAI-1 in diabetic renal fibrosis [19,20,21,23,24]. HG-induced upregulation of PAI-1 in MCs further supports the findings from prior in vitro and in vivo studies [28,83]. GYY treatment restored PAI-1 to basal levels, comparable to controls, both in vitro and in vivo. A previous study suggested that the inhibition of PAI-1 reduced ECM accumulation and renal fibrosis [25]. Collectively, our findings indicate that GYY treatment may be effective in preventing PAI-1-mediated ECM remodeling and thereby demonstrate renoprotection during diabetes.

MMPs are crucial regulators for ECM homeostasis in the kidney [84]. Previous reports demonstrated that the upregulation of MMP-9 and MMP-13 results in adverse diabetic renal remodeling [12,13,85]. The present study corroborates findings from our and others’ previous studies [12,13,86]. In accordance with in vivo observations, in vitro experiments revealed that HG induced the expression of MMP-9 and MMP-13 in MCs. GYY treatment alleviated elevated expression in diabetic kidney and HG-exposed MCs, reinforcing the previous reports [12,13]. Taken together, our findings not only indicate an important role of MMP-9 and MMP-13 in the ECM accumulation, but also suggest the functional relevance of MMP regulation by exogenous H_2_S supplementation in diabetic kidney. 

The major factor contributing to the pathological features of renal fibrosis is the dysregulation of ECM proteins in diabetic kidney [13]. In DN, the major ECM proteins that accumulate are collagen, fibronectin and laminin [87,88,89]. PAI-1 is known to regulate the synthesis and degradation of the ECM proteins, fibronectin and laminin [15], whereas MMP-9 predominantly cleaves collagen IV, the major component responsible for the expansion of mesangial matrix and glomerular basement membrane thickening [84]. A previous study conducted in a hypertensive rat model showed that elevated renal expression of PAI-1 and MMP-9 resulted in kidney fibrosis [90]. To investigate the consequences of upregulated PAI-1, MMP-9 and MMP-13, we measured the levels of major ECM proteins. We observed a distinct upregulation of Col IV and fibronectin expression in the diabetic kidney and MCs under HG conditions, which was mitigated following GYY treatment. It is noteworthy that our previous study demonstrated increased collagen accumulation in the peri-glomerular and glomerular space of the diabetic kidney. In the present study, collagen accumulation was predominantly seen in the glomerular region, similar to earlier studies [12,13]. Despite a plethora of evidence that there was increased MMP-9 and Col IV expression in both in vitro and in vivo diabetic models, it is not clear how elevated MMP-9 contributes to excessive collagen accumulation in DN. Previously, our group suggested that the downregulation of miR-194 induces MMP-9 and Poly ADP ribose polymerase 1 (PARP-1), which, in turn, facilitate Col IV upregulation, leading to peri-glomerular collagen deposition in the type-1 diabetic kidney [13]. Although elevated MMP-9 degrades normal collagen IV, it is ineffective in cleaving collagen IV modified by nonenzymatic glycation, leading to abnormal collagen accumulation, and thus, glomerular basement membrane thickening [72,91,92]. Moreover, MMP-9 triggers TGF-β-induced epithelial–mesenchymal transdifferentiation (EMT), which contributes to subsequent collagen deposition in the interstitial space [93,94,95]. In the current study, we also noticed elevated fibronectin and laminin expression in the glomeruli of the type-1 diabetic kidney. Moreover, our in vitro findings support previous reports that HG promotes the accumulation of major ECM proteins such as collagen, fibronectin, etc., in mesangial cells [96,97]. In addition, it is worth mentioning that earlier studies from our laboratory and others also showed that H_2_S supplementation ameliorates elevated collagen and fibronectin expression in the diabetic kidney [2,6,12,13,50]. In DN, elastin degradation is considered to be an important contributor to ECM accumulation, and thus, renovascular remodeling. Previous studies from our laboratory demonstrated increased degeneration of perivascular elastic fibers in the diabetic kidney, whereas H_2_S supplementation substantially abrogated the vascular pathology [12]. In accordance with this previous finding, our present study showed a marked decrease in elastin expression in the diabetic kidney and HG ambient MCs, whereas the upregulation of elastin levels following GYY treatment suggested the potential role of GYY in preventing the formation of fibrovascular tissue in the diabetic kidney. Collectively, our present study strongly suggests that elevated PAI-1 levels in the diabetic kidney and HG-exposed MCs is involved in the upregulation of MMP-9 and MMP-13, leading to increased ECM turnover, which was substantially ameliorated by GYY treatment.

PAI-1 has been shown to be upregulated in HG-exposed glomerular mesangial cells [28,29] and also in excessive ECM accumulation and renal fibrosis during DN [22,23]. Our results further support these earlier findings. Recently, reactive oxygen species (ROS) have been identified as an important mediator of the HG-induced elevated expression of PAI-1 in MCs and diabetic kidney [83]. However, the signaling pathways involved in the hyperglycemia- or HG-induced upregulation of PAI-1 are yet to be defined [83]. Previously, retinoid (tRA and 9-cis RA)-induced upregulation of PAI-1 was evident in cultured smooth muscle cells [98]. Recently, the induction of RXR and RAR was shown to exert pro-fibrotic effects in the renal fibroblast under the influence of retinoids (tRAs) as well as by pan-RXR and pan-RAR agonists [14]. Furthermore, these pro-fibrotic effects were associated with increased PAI-1. Thus, involvements of RAR and RXR are suggested as crucial mediators in the PAI-1-dependent fibrogenic effect in renal fibroblasts [14]. In the present study, we observed upregulations of RXRα, RXRβ and RARγ1 in HG ambient MCs and diabetic kidney, which were also associated with increased PAI-1. Therefore, our results suggest the involvement of RAR and RXR in PAI-1-dependent ECM turnover in MCs under HG and diabetic kidney. Treatment with GYY substantially mitigated the elevated expression of RXRα, RXRβ and RARγ1, suggesting an RXR and RAR regulatory role by GYY to mitigate PAI-1, MMP-9, MMP-13; thus, ECM turnover in diabetic kidney. It has been already discussed that both RARs and PPARs can heterodimerize with RXR to regulate the expression of their target genes by binding to specific promoters [37,47,48]. In this context, it is worth mentioning that the possible role of PPARγ agonist-mediated heterodimerization of PPARγ and RXR is suggested to mitigate MMP-9-induced DN [82]. Therefore, our study suggests that PPARγ antagonism and RARγ1 agonism may contribute to the induction of PAI-1, MMP-9 and MMP-13, possibly via RXR signaling, whereas GYY treatment attenuated adverse ECM turnover by modulating PPAR/RAR-mediated RXR signaling. Moreover, it is noteworthy that the ‘STITCH 5.0′ protein–protein interaction network provides strong evidence in support of the potential involvement of PPAR/RAR-mediated RXR signaling in the regulation of PAI-1-dependent ECM turnover. In addition, protein–chemical interactions suggest a strong association of PPARγ agonist (rosiglitazone) as well as the RAR/RXR agonist (retinoic acid) in the possible regulatory role of RXR signaling in ECM turnover indicating another scope for future treatment strategies of DN by using combinatorial therapy involving PPAR/RAR/RXR agonists and H_2_S supplementation. The present study demonstrates a potential role of GYY-mediated regulation of the RXR signaling pathway to ameliorate adverse ECM turnover in the diabetic kidney. However, further studies are required to explore the mechanistic insight of GYY therapy on multifaceted crosstalk among PPAR, RAR and RXR in the diabetic scenario.

In summary, the present study demonstrates that in type-1 diabetic kidney, hyperglycemia results in the downregulation of PPARγ and the upregulation of RXRα, RXRβ and RARγ1, which possibly contributes to elevated PAI-1 levels, and thus, elevated MMP-9 and MMP-13. Together, it results in the increased expression of Col IV, fibronectin, laminin and decreased elastin, leading to ECM remodeling in the diabetic kidney. H_2_S supplementation by GYY ameliorates excessive ECM accumulation by restoring normal levels of PAI-1, MMP-9 and MMP-13 via the modulation of PPAR/RAR-mediated RXR signaling. In addition, our in vivo observations were further validated by in vitro study wherein high-glucose-induced diminished H_2_S production exerts pathological changes similar to that of diabetic kidney in murine mesangial cells, which is ameliorated by GYY treatment. Therefore, our study shows, for the first time, that the regulation of RXR signaling by GYY may be useful as a potential therapeutic target to ameliorate PAI-1-dependent adverse ECM turnover in type-1 DN. However, in-depth mechanistic studies are required for better understanding of our initial findings prior to the successful therapeutic manipulation of RXR signaling by GYY in the treatment of DN in future.

## Figures and Tables

**Table 1:** Sequences of Primers.

MMP-9	Forward 5′-CACACGACATCTTCCAGTACCA-3′ Reverse 5′-TCATTTTGGAAACTCACACGCC-3′
MMP-13	Forward 5′-CAGTTGACAGGCTCCGAGAA-3′ Reverse 5′-TTCACCCACATCAGGCACTC-3′
Col IV	Forward 5′-GACCACTATGCTTGAAGTGA-3′ Reverse 5′-ACAGAAGGCCTTAGTAGTCT-3′
Fibronectin	Forward 5′-TTGTTCGGTGGAGTAGACCC-3′ Reverse 5′-TTCAGGGAGGTTGAGCTCTG-3′
Elastin	Forward 5′-TGACAGTATAGGGCTGAGCA-3′ Reverse 5′-GAGTTGTTGTGGGTGAGACA-3′
GAPDH	Forward 5′-GTCAAGGCCGAGAATGGGAA-3′ Reverse 5′-GGCCTCACCCCATTTGATGT-3′

## Data Availability

The data presented in this study are available upon request from the corresponding author.
